# Intradiscal Injection of Autologous Discogenic Cells in Patients with Discectomy: A Prospective Clinical Study of Its Safety and Feasibility

**DOI:** 10.1093/stcltm/szac013

**Published:** 2022-04-15

**Authors:** Anwu Xuan, Dike Ruan, Chaofeng Wang, Qing He, Deli Wang, Lisheng Hou, Chao Zhang, Chao Li, Wei Ji, Tianyong Wen, Cheng Xu, Zhenbiao Zhu

**Affiliations:** 1 The Second School of Clinical Medicine, Southern Medical University, Guangzhou, People’s Republic of China; 2 Department of Orthopedics, The Sixth Medical Center of PLA General Hospital, Beijing, People’s Republic of China; 3 Department of Orthopedics, Xi’an Honghui Hospital, Xi’an, People’s Republic of China; 4 Department of Orthopedics, Peking University Shenzhen Hospital, Shenzhen, People’s Republic of China

**Keywords:** intervertebral disc degeneration, cell transplantation, discogenic cells, discectomy, lumbar disc herniation

## Abstract

The treatment of intervertebral disc degeneration (IVDD) is still a huge challenge for clinical updated surgical techniques and basic strategies of intervertebral disc regeneration. Few studies have ever tried to combine surgery and cell therapy to bridge the gap between clinical and basic research. A prospective clinical study with a 72-month follow-up was conducted to assess the safety and feasibility of autologous discogenic cells transplantation combined with discectomy in the treatment of lumbar disc herniation (LDH) and to evaluate the regenerative ability of discogenic cells in IVDD. Forty patients with LDH who were scheduled to have discectomy enrolled in our study and were divided into the observed group (transplantation of autologous discogenic cells after discectomy) and control group (only-discectomy). Serial MRI and X-ray were used to evaluate the degenerative extent of index discs, and clinical scores were used to determine the symptomatic improvement. No adverse events were observed in the observed group, and seven patients in the control group underwent revisions. Both groups had significant improvement of all functional scores post-operatively, with the observed group improving more considerably at 36-month and 72-month follow-up. The height and water content of discs in both groups decreased significantly since 36 months post-op with the control group decreased more obviously. Discectomy combined with autologous discogenic cells transplantation is safe and feasible in the treatment of LDH. Radiological analysis demonstrated that discogenic cells transplantation could slow down the further degeneration of index discs and decrease the complications of discectomy.

## Graphical Abstract

**Figure:**
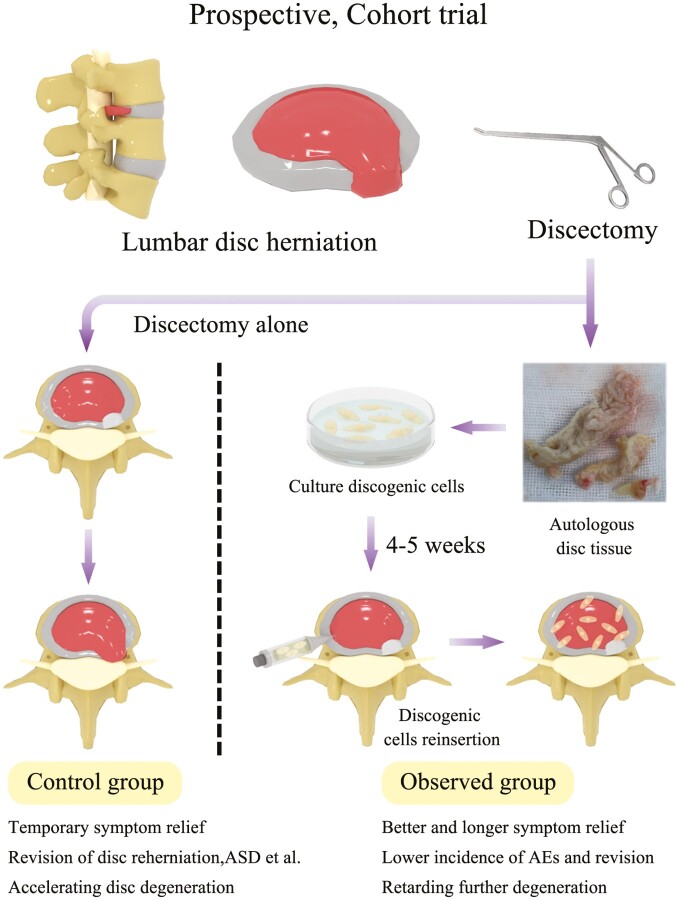
Autologous discogenic cells transplantation could potentially compensate for the shortcomings of discectomy by retarding the intervertebral disc degeneration.

**Lessons Learned**

- Discectomy combined with autologous discogenic cells transplantation is safe and feasible in the treatment of intervertebral disc degeneration.
- Beneficial outcomes favoring cell transplantation, reduced pain, and increased function.
- Radiological analysis showed that autologous discogenic cells transplantation could slow down the further degeneration of index discs after discectomy.

**Significance Statement**

This is the first prospective, cohort trial with long-term follow-up to evaluate the regenerative restoration ability of autologous discogenic cells in intervertebral disc degeneration. Autologous discogenic cells transplantation may have potential advantages over other monotype cell therapy repairing disc degeneration. Surgery in conjunction with biological therapy would be regarded as a novel concept to achieve symptom relief and prevention of secondary disease simultaneously.

## Introduction

Intervertebral disc degeneration (IVDD), a normal, unidirectional, and irreversible process with aging, is a predominant source of low back pain and has a significant socioeconomic impact given the associated disability.^1^ Low back pain is the most common clinical manifestation in the process of IVDD and is now the leading cause of disability globally.^2^ Lumbar disc herniation (LDH), commonly considered an extension of progressive disc degeneration, frequently comes with sciatica, numbness, and claudication in addition to low back pain.^3^ Intervertebral discs are the root or culprit of all these health problems.

The intervertebral disc is a special anatomical structure located between 2 vertebrae and accounts for one-third of the total human spine length, absorbing and distributing complex loads, and providing spinal stability while permitting motion. Macroscopically, the intervertebral disc is a special complex composed of a gel-like core-nucleus pulposus (NP), which is laterally encapsulated in layers of annulus fibrosus (AF) and sandwiched by 2 thin layers of cartilage endplate (CEP). Microscopically, these tissues consist of different but interrelated cell populations and specific matrix structures.^4^

Despite the morphological differences in the structural organization of the different anatomical components of the IVD to which they belong, NP cells, AF cells, and CEP cells are closely interdependent and play a crucial role in maintaining the integrity and biomechanical function of the intervertebral disc.^4^ However, in the process of disc degeneration, degenerative changes in the biomechanical and structural properties of these 3 kinds of cells usually occur concurrently, resulting in imbalanced homeostasis, decreased cells and extracellular matrix (ECM), reduced intradiscal pressure, and reduced nutritional supply. All these domains consist of a vicious circle of disc degeneration, potentially inducing disarrangements such as intervertebral disc herniation, spondylolisthesis, and spinal stenosis.^5^

LDH is the most frequent type of disc degeneration and the most common cause of radicular or sciatic pain. However, approximately 60%-90% of patients with LDH are mild and generally managed with conservative interventions, eg, physical therapy, medication, or block therapy. For the remaining patients at a severe stage or with refractory to conservative treatments for at least 6 months, surgical intervention should be considered.^6,7^ With the continuous development and progress of surgical technology, there have been many surgical techniques for LDH, ranging from discectomy to lumbar fusion to total disc replacement (TDR) and minimally invasive surgery under endoscopes.^8^ Surgical techniques are very effective for temporary neurological decompression by removing herniated intervertebral discs and partly eliminating chemical stimulation simultaneously in patients suffering from LDH. However, these surgical interventions remain highly controversial for their long-term efficacy and complications. Discectomy removes the disc tissue that compresses the nerve but destroys the integrity of the annulus fibrosus, resulting in 5.2% reherniation and 37.5% recurrence of sciatica.^9^ Lumbar fusion could remove the disc tissue completely at the index level with the sacrifice of segmental motion, leading to 36% postoperative complications, 27% revision,^10^ and 82.6% adjacent segment degeneration.^11^ Although TDR can partially preserve segmental mobility, the clinical effect is equivalent to that of fusion surgery, and the incidence of symptomatic adjacent disease after surgery is also as high as 34%.^12,13^ Minimally invasive surgery relieves the pain of patients with minimal trauma, but the incidence of disc reherniation is even higher than that of discectomy, and there is a higher risk of incomplete decompression.^8,14^ The root cause of all these consequences is that these surgeries do not intend to restore the IVD but to accelerate the degeneration process.

Against this backdrop, advancements in regenerative medicine have led to an effervescence of growth in the development of various experimental and preclinical trials aiming at restoring or re-establishing a healthy IVD. The 3 main methods are cell therapy, growth factor therapy, and gene therapy. Cell therapy is currently the most studied and has made major breakthroughs and successes in animal experiments and preclinical experiments and has gradually advanced from the lab to the clinic. It will likely fill the gap between conservative therapy and surgeries for the treatment of IVDD.^15^

However, current clinical studies focus primarily on the safety and short-term efficacy of different types of cells and are still within the pilot or phase I stage without control groups. Moreover, most of these studies choose moderate disc degeneration as the indications for which it would be improved by conservative treatments. Although cell therapy represents a potentially low-risk and low-cost solution to address the tremendous unmet need for new treatment options for patients with disc degeneration, it is disadvantageous in treating symptomatic LDH, especially in neural decompression. Cell therapy has not yet been clinically studied for disc repair after discectomy and for preventing accelerated disc degeneration. Although these treatments have used a variety of cell types, including autologous or allogeneic cells obtained from notochordal,^16^ chondrocyte-like cells,^17^ and mesenchymal stromal cells (MSCs),^18^ only a monotype of cell is used to repair the intervertebral disc, which is a complex composed of 3 kinds of cells in clinical research. Theoretically, these cells, which were transplanted into the discs, possess multiple proliferative capacities, but the hostile microenvironment in the degenerated IVD hallmarked by low cellularity, hypoxia, acidic conditions, and mechanical loading remains the greatest challenge for transplanted cells to remain viable for a long time, not to mention for functioning well. There is still a long way to go to overcome these difficult problems, although the following solutions have been attempted: preconditioning the cells under the mimicking milieu of discs,^19^ activating the cells by cell-to-cell coculture,^20^ and combining them with growth factors or scaffolds.^21,22^ Autologous IVD-derived cell transplantation seems to be more natural physiologically and can avoid the risk of graft-versus-host reactions and ethical issues. More importantly, IVD-derived cells would be more capable of adapting to the harsh microenvironment and remain viable longer. This method dates back to 1998, and Nishmura and Mochida first reported that percutaneous reinsertion of autologous normal NP tissue could delay disc degeneration in a rat model.^23^ In 2002, the EuroDISC trial was initiated by Meisel and his colleagues.^24-26^ In their interim analysis, they found that autologous disc chondrocyte transplantation (ADCT) was technically feasible and biologically relevant to repairing disc damage and retarding disc degeneration. Later, Tschugg et al^27,28^ also gave their short report of the ongoing ANVOVART disc plus a study to assess the safety and efficacy of ADCT plus a hydrogel in the repair of herniated nucleotomized discs, but no specific results have been reported yet. Overall, although these studies used ADCT, the cells they used were actually only NP cells, as in the study of Mochida et al,^20^ which may influence the interaction of the 3 kinds of disc cells in the regeneration process and in the repair of the whole disc.

Taken together, we hypothesize that discogenic cells, ie, IVD-derived cells consisting of NP cells, AF cells, and CEP cells, would be the optimal kinds of cells to repair the disc. The optimal resource of discogenic cells is undoubtedly the disc tissues removed from discectomy in the treatment of disc herniation. These disc materials can be part of the NP, annulus fibrosus, endplate material, or a combination of the above. Moreover, our previous studies^29,30^and other studies^31-34^ have demonstrated the presence of progenitor cells in the tissue of the IVD and compared the properties of IVD progenitor cells with MSCs. Lyu et al^5^ reviewed these studies and pointed out that IVD progenitor cells might have superior potential to non-IVD-derived MSCs for IVD cell differentiation and be better able to adapt to and engraft into host IVD tissue after transplantation. Together, transplantation of discogenic cells, which may consist of 3 kinds of cells and their progenitor cells, would potentially restore and repair the disc complex as a whole.

For patients with symptomatic disc herniation scheduled for discectomy, culturing the discogenic cells from the removed disc tissue and reinserting them into the index levels would take full advantage of both surgery and cell therapy, including symptom relief and prevention of accelerating degeneration simultaneously. However, no clinical study hitherto has assessed the long-term efficacy of this combined strategy.

In this study, we evaluated the safety and feasibility of discectomy followed by autologous discogenic cell transplantation in the treatment of LDH and the regenerative restoration ability of discogenic cells in clinical outcomes with long-term follow-up.

## Materials and Methods

### Study Design

**Figure 1.**
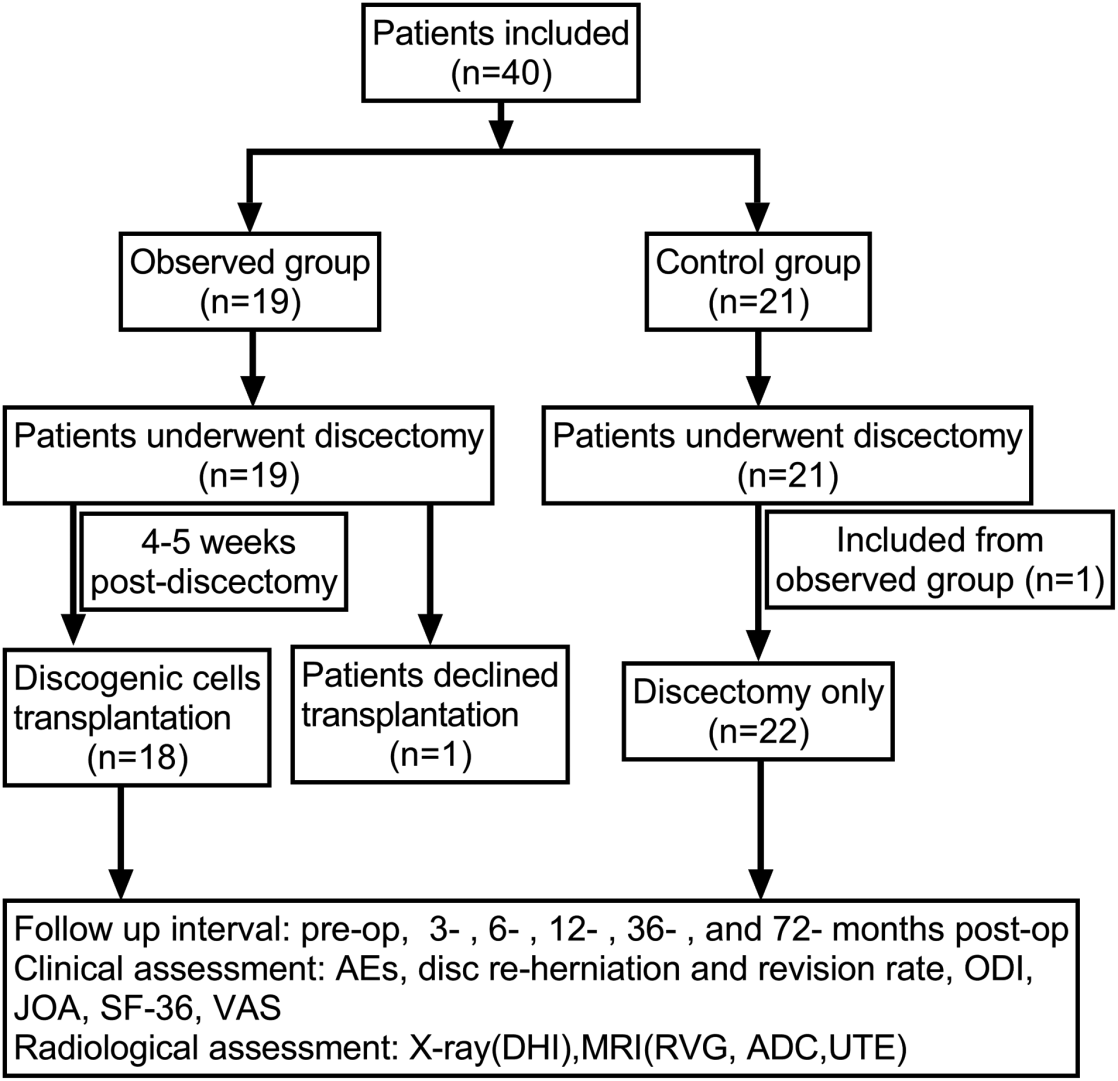
The flow chart of the study design.

This study was a prospective open-label, nonrandomized controlled clinical trial performed at a single center. Prior to undertaking the study, ethical clearance was obtained from the Ethics Committee of the Sixth Medical Center of PLA General Hospital (Beijing, China). All patients received an explanation of the project and signed the approved informed consent before the intervention. Each patient participating in the clinical trial underwent surgical treatment for their disc prolapse, and according to the patients’ willingness, those included in the observed group would accept the subsequent cell transplantation. The remaining patients who underwent only discectomy were classified as the control group. The flow-chart of our study is shown in Fig. 1.

## Study Population

### Patient Selection

**Figure 2.**
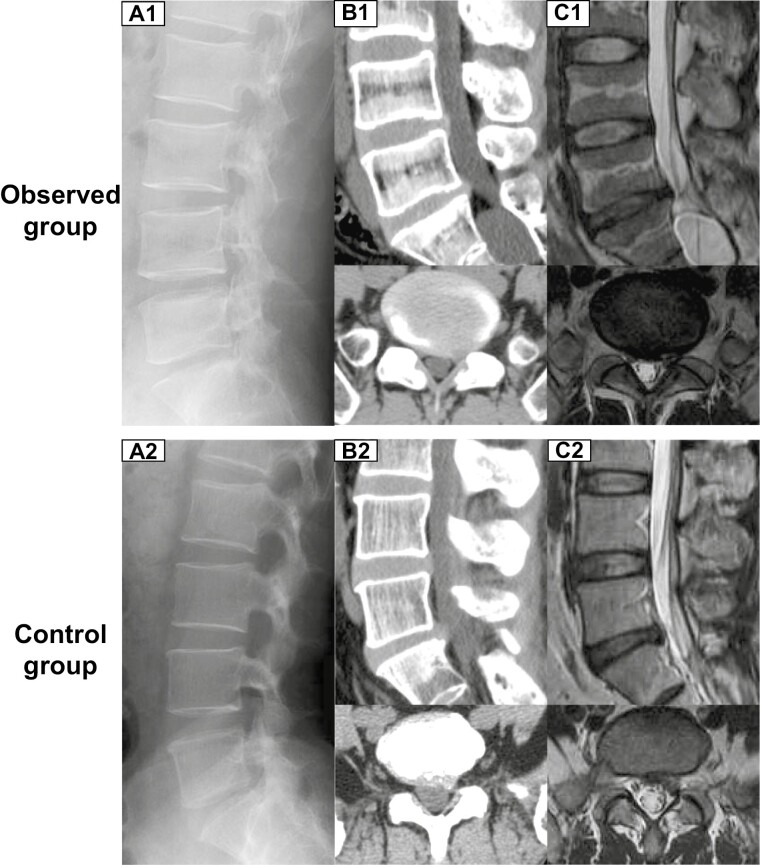
Preoperative images of both groups. A1-C1 shows X-ray, CT, and MRI of 1 patient in the preoperative observation group. A2-C2 shows X-ray, CT, and MRI of 1 patient in the preoperative control group.

We included participants meeting the following inclusion criteria (Table 1) who had been diagnosed with disc herniation and confirmed clinically and radiologically and who were scheduled for lumbar discectomy between May 2013 and September 2015 and then divided into the observed group (discectomy combined with cell transplantation) and the control group (discectomy alone) according to their choices. Preoperative images of both groups are shown in Fig. 2. Patient demographic information, such as age, sex, and body mass index (BMI), was recorded after enrollment, and all the participants signed confidentiality agreements.

**Table 1.** Inclusion and exclusion criteria.

Inclusion criteria	Age between 18and-60 years
	Single-level lumbar disc herniation confirmed on MRI that was consistent with history and physical examination
	Clinical diagnosis of low back pain and/or sciatica
	Failed conservative treatment (ie, physical therapy, medications, epidural injection,) for a minimum of 6 months
	Physically and mentally able to participate in the study and the follow-up visit and willing to undertake the possible risk factors involved
Exclusion criteria	Previous surgery at the lumbar spine
	Disc re-herniation treated with nucleotomy/ sequestrectomy of the relevant disc
	Degenerative changes in the lumbar as determined by Modic Changes 2 or 3/Pfirrmann 4 or 5
	Segment instability (spondylolisthesis >5 mm or translation >3 mm)
	Lumbar scoliosis or kyphosis
	Previous compression or burst fracture at the level to be treated
	Severe lumbar stenosis with evidence of a narrowing of <8 mm (by sagittal MRI)
	Spinal tumor
	Body mass index> 30 kg/m^2^
	Pregnant, breastfeeding, or planning to become pregnant within 2 years
	Unable to undergo MRI test
	Immune defects or immune suppression
	Active systematic or local infections
	Severe cardiac disease, pulmonary disease, active neoplasm, anemia, or any other surgical contraindications

### Surgical Procedure

All patients were placed in a prone position after general anesthesia. The level of the discectomy was verified using intraoperative fluoroscopy. A posterior middle-line approach was used, and back muscles were detached from the spinous process and retracted unilaterally. Fenestration was used as the caudal part of the cephalad lamina, and the ligamentum flavum was removed to expose the compressed nerve roots and herniated disc. Only herniated disc material and a small portion of the intervertebral disc were removed. The disc tissues in the control group were abandoned as usual, but the tissues in the observed group were collected and sent to the GMP lab for cell culture.

### Cell Production and Preparation

Discogenic cells were isolated as described in our previous studies.^29,30^ Briefly, disc tissues were harvested during discectomy and transferred to the GMP lab for culturing. Disc tissues were washed 3 times with PBS. Vessels and ligaments surrounding the disc were removed as much as possible, but AF, NP, and CEP were retained, and disc samples were fragmented into no larger than 1-mm^3^ pieces with ophthalmic scissors. Subsequently, tissue fragments were digested with 0.2% (m/v) collagenase II (Sigma-Aldrich; Merck KGaG, Darmstadt, Germany) for 6 hours at 37 °C. Primary cells were obtained after filtering through cell strainers with a pore size of 70 µm, suspended in Dulbecco’s modified Eagle’s medium-low glucose supplemented with 10% fetal bovine serum (both from Sigma-Aldrich; Merck KGaG), and then seeded in 25-cm^2^ flasks at a density of 1 × 10^5^/mL. Cells were cultured in an incubator at 37 °C in a humidified atmosphere containing 5% CO_2_ and passaged upon reaching 80% confluence. The medium was changed every 3 days, and discogenic cells at passage 3 were resuspended in Dulbecco’s modified Eagle’s medium at a density of 2 × 10^6^ cells/mL). Before transplantation, the cells were harvested, washed twice, and resuspended in physiologic saline at a target volume for transplantation. The supernatant media were sent to the Department of Laboratory to test for bacterial, fungal, mycoplasma, and endotoxin contamination. Cultured cells were placed in a 5-mL disposable syringe, packaged aseptically, and brought to the operating room in an ice chest.

### Cell Transplantation

Cell transplantation was conducted 4-5 weeks after discectomy to ensure that the annulus had healed and contained the cells. Once discogenic cells had been subcultured and suspended in normal saline, they were prepared for reinjection in a syringe. Patients were brought into the operation room and placed in a prone position on an operating room table, and after standardized sterile preparation, the injection site was treated with local anesthetic (1% buffered lidocaine). Using a pressure-volume test prior to the delivery of cells, cells could be placed with confidence that they would be retained at the site of delivery. Discogenic cells were percutaneously injected into the index disc on the opposite side from the dicectomy through a standard posterior lateral discogram approach over the superior articular process, with the starting point being as lateral as possible to allow as much of the injectate as possible to be injected into the posterior disc annulus with a 22-gauge needle under fluoroscopic guidance. Approximately 1-3 mL (4-7) × 10^6^ of the cultured cells were injected into the symptomatic lumbar disc. The needle remained in place for 3-5 minutes after injection to prevent cell leakage. Patients were prescribed pain medicine to be used as needed for 1-7 days and placed on restrictions as tolerated. Physical therapy postinjection was not restricted but was encouraged.

### Follow-Up Protocol

All patients were discharged postdiscectomy between the seventh and 12th days. Clinical evaluation and routine analysis were conducted for all the patients, and the observed group of patients needed extra cell transplantation. At 3, 6, 12, 36, and 72 months after discectomy, the patients were scheduled for outpatient clinic appointments via phone and/or email. They were clinically examined during their follow-up and followed with radiological imaging. Any participants who underwent a secondary fusion operation at any time terminated the later follow-up.

### Clinical Assessment

The safety assessments mainly contained adverse events (AEs), disc reherniation and revision related to the intervention during treatment and follow-up. The clinical efficacy involves the following scoring systems: the visual analog scale (VAS) was used to assess changes in leg and low back pain and the Oswestry Disability Index (ODI) was used to assess the limitations of various activities of daily living.^35^ The Short Form Health Survey-36 (SF-36)^36^ questionnaire and Japanese Orthopedic Association (JOA) scoring system for low back pain^37^ were applied to assess changes in health status and quality of life.

### Radiological Assessment

Radiological examinations were used to evaluate the regenerative ability of discogenic cells indirectly, including X-ray to measure the disc height and MRI to evaluate the hydration condition of the index discs.

**Figure 3.**
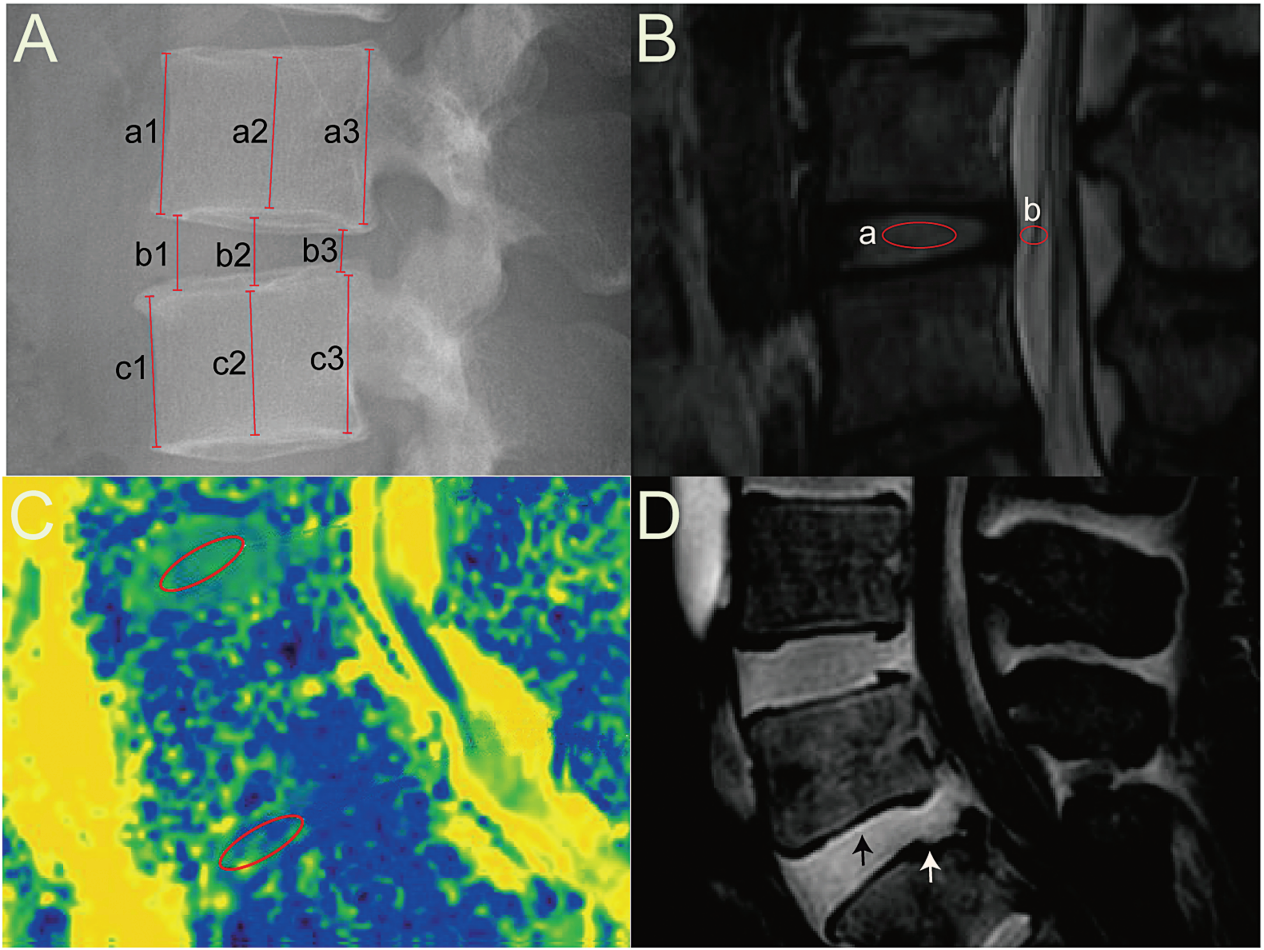
Radiological measurement (**A)** disc height index (DHI); a1-a3, b1-b3, and c1-c3 represent the anterior, middle, and posterior heights of the disc and cephalic and caudal vertebrae, DHI = 2 × (b1 + b2 + b3)/[(a1 + a2 + a3) + (c1 + c2 + c3)]. (**B**) Ratio value of the grayscale (RVG). Midsagital T2-weighted images were chosen, and RVG was the grayscale of discs (a) normalized against the grayscale of cerebrospinal fluid at the same level (b). (**C**) Measurement of the apparent diffusion coefficient (ADC) on diffusion-weighted images (DWI) in the index discs (inferior region of interest, ROI, 40 mm^2^) and the adjacent normal discs (superior ROI, 40 mm^2^). (**D**) MRI sequence of sagittal ultrashort time to echo (UTE) illustrating the hyperintensity of CEP with continuity of high signal of integral CEP (Black arrow) on the cranial side and discontinuity of the high signal of CEP defect (white arrow) on the caudal side.

Disc height: IVD height and vertebral height were converted to the disc height index (DHI) according to the methods applied by our previously published protocols.^38^ In short, the intervertebral DHI was obtained by calculating the average values from a medial plane at 3 points of the posterior, middle, and anterior parts of the involved IVD, and these values were divided by the average height of the neighboring vertebrae. DHI was determined as 2× ((b1 + b2 + b3)/(a1 + a2 + a3 + c1 + c2 + c3)) × 100% (Fig. 3A).

Water content of discs: 3.0TMRI (Philips, Medrad Spectris Solaris EP, USA) was performed to obtain midline T2-weighted sagittal images of index discs. The severity of IVDD at each follow-up was assessed using Pfirrmann’s classification.^39^ We used Photoshop version 2020 (Adobe, San Jose, CA, USA) to measure the grayscale of discs and cerebrospinal fluid at the same level. The grayscale of the disc was normalized against that of the cerebrospinal fluid and represented as the ratio value of the grayscale (RVG) (Fig. 3B). At the last follow-up, apparent diffusion coefficient (ADC) mapping from the diffusion-weighted coefficient (DWI) was performed on 6 patients in each group to quantitatively determine changes in water content in the index discs compared with that of normal adjacent discs. DWI data measurement was performed on an imaging workstation (Philips Extended MR WorkStation). First, we selected an appropriate region of interest (ROI) to measure the ADC value of the affected disc according to its size. Then, the same ROI was used to measure the ADC value of adjacent normal segments. Finally, the 2 values were used to normalize the ADC value of the index level (Fig. 3C). We also conducted ultrashort time-to-echo (UTE) MRI in these patients to evaluate the integrity of the CEP of index discs. CEP defects were defined as discontinuity of the high signal on UTE MRI^40^ (Fig. 3D). All MRI scanning parameters are shown in Table 2.

**Table 2.** MR scanning parameters.

Sequence	TR (ms)	TE (ms)	Slice (mm)	Flip angle (degree)	FOV (mm)	AP(freq.)*FH(phase)	Recon matrix	Gap (mm)	NSA
T2WI	3000	120	4	90	250	200 × 200	252 × 191	0.4	2
DWI	3000	106	8	90	96	96 × 96	76 × 73	8	1
UTE	4	0	4	15	200	200 × 200	200 × 200	−2	1

T2WI, T2-weighted imaging; DWI, diffusion-weighted images; UTE, ultrashort time to echo; TR, repetition time; TE, echedelay time; FOV, field of view; AP, anterior and posterior; FH, foot and head; NSA, number of signal average.

All of the data measurements were performed 3 times by 2 independent assessors without knowledge of the clinical information, and the mean of these values was taken for analysis.

All data were transferred to Microsoft Excel (Microsoft, Redmond, Washington, DC, USA) and graphically presented by GraphPad Prism (GraphPad Inc., San Diego, CA, USA).

### Statistical Analysis

The measurement data are presented as the means ± SD, and the counting data are expressed as percentages (%). Pearson’s chi-square, Fisher’s exact test, Student’s *t* test, the Mann-Whitney *U* test, or Wilcoxon signed-rank test was used to analyze the within- and between-group differences at baseline and post-treatment of each group for the clinical scores and radiological measurements. Repeated-measures analysis of variance was used to compare the significant differences between the groups at each time point. Patients without baseline scores were excluded from the respective analyses. Missing data (due to incomplete patient interviews, study withdrawal) were filled with a multiple imputation method to enable individuals with incomplete data to be included in the analysis. All data processing was performed utilizing IBM SPSS Statistics 26.0.

## Results

### Demographics

Forty patients met the inclusion criteria and were followed in the treatment registry. Out of 19 patients in the observed group, one subject refused to accept cell transplantation after discectomy and was included in the control group.

In the observed group (*n* = 18), 16 (88.9%) patients were males, and 2 (11.1%) were females, with a mean age at surgery of 36.39 years (SD = 11.93; min = 22; max = 59). BMI was averaged at 24.68 kg/m^2^. Their duration of symptoms ranged from 6 to 28 months. All patients were treated with a single-level discogenic cell transplantation following discectomy, with the L5-S1 level treated most commonly (10/18; 55.56%), followed by the L4-L5 IVD (8/18; 44.44%). The average cell dose was 5.57 × 10^6^ cells/disc, and the mean cell viability was 93.23%.

In the control group (*n* = 22), 16 (72.7%) patients were males, and 6 (27.3%) were females, with a mean age at surgery of 40.73 years (SD = 12.01; min = 21; max = 59). BMI was averaged at 24.79 kg/m^2^. Their duration of symptoms ranged from 7 to 33 months. All the patients were only treated with single-level discectomy, with 12 (54.55%) L5-S1 levels, 9 (40.90%) L4-L5 levels, and 1 (4.55%) L3-L4 levels. There was no significant difference in the baseline data between the 2 groups (*P* > .05). The patient details are summarized in Table 3.

**Table 3.** Patient characteristics.

**Demographics**	**Observed group (*n* = 18)**	**Control group (*n* = 22)**	**Statistical value)**	***P*-value)**
Age at surgery (year)	36.39 ± 11.93	40.73 ± 12.01	*t* = −1.14	*P* = .261
Gender			Fisher’s exact test	*P* = .258
Male	16 (88.9%)	16 (72.7%)		
Female	2 (11.1%)	6 (27.3%)		
Operation level			□χ^2^ = 2.939	*P* = .203
L3/4	0	1 (4.55%)		
L4/5	8 (44.44%)	9 (40.90%)		
L5/S1	10 (55.56%)	12 (54.55%)		
BMI(kg/m^2^)	24.68 ± 3.21	24.79 ± 2.60	*t* = −0.111	*P* = .912
Preoperative Pfirrmann grade			*U* = 160.50	*P* = .227
II	5 (27.78%)	9 (40.90%)		
III	13 (72.22%)	12 (54.55%)		
IV	0	1 (4.55%)		
Symptom duration (months)	14.28 ± 6.48	16.23 ± 7.01	*t* = − 0.905	*P* = .371
Cell number ((×10^6^))	5.75 ± 0.60	None		
Cell viability (%)	93.23 ± 2.93	None		
Follow-up time (median/months)	84	72	*U* = 157.50	*P* = .274

BMI, body mass index.

### Clinical Outcomes

#### Safety Variables

**Figure 4.**
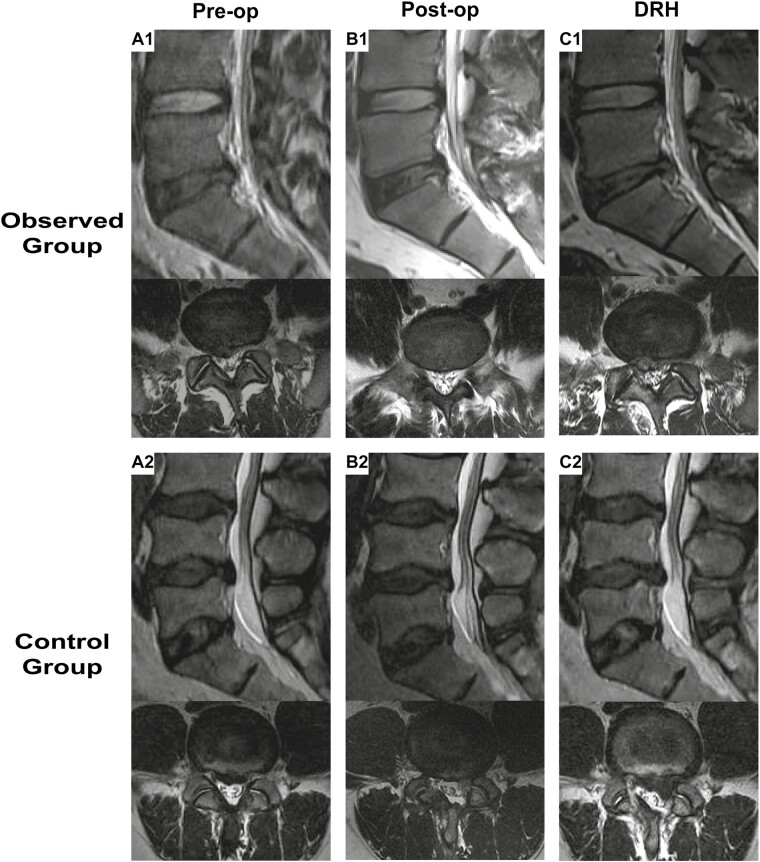
Disc reherniation after the operation in the 2 groups. A1 shows the L5/S1 herniation of one patient in the preoperative observation group. B1 shows the index level 3 months postoperatively. C1 shows reherniation 72 months postoperatively. A2 shows the L4/5 herniation of one patient in the control group preoperatively. B2 shows the index level 3 months postop. C2 shows reherniation 11 months postop. Preop, preoperation; Postop, postoperation; DRH, disc reherniation.

In the observed group, no patients suffered from immunoreactions, such as fever, allergic reaction, or local infection, from immediately after cell transplantation to 3 months postoperation. Two subjects (11.11%) experienced low back pain at 1 to 2 years postoperation, which was relieved by medication. One patient (5.56%) developed disc reherniation 72 months after the operation but refused to undergo secondary surgery. In the control group, 9 patients (36.36%) had low back pain and sciatica during the follow-up, of which 7 patients (31.82%) progressed to revision during the follow-up due to the following reasons: 4 patients (18.18%) elected to have surgery because of disc reherniation within 36 months postoperatively (11, 21, 28, and 35 months), 2 patients (9.09%) elected to have surgery because of lumbar canal stenosis of the index level between 48 and 72 months (50 and 68 months), and one patient (4.55%) elected to have surgery because of spondylolisthesis 56 months postoperatively. The total number of revisions (*n* = 0) in the observed group was significantly lower than that in the control group (*n* = 7), *P* = .04 (Table 4; Fig. 4).

**Table 4.** Comparison of patients’ complications during the follow-up.

**Complications**	**Observed Group (n=18)**	**Control Group (n=22)**	**Statistical value**	***P* value**
Low back pain or Lower limbs pain	3 (16.67%)	9 (40.90%)	χ^2^=5.560	*P* = .035
Disc re-herniation	1 (5.56%)	4 (18.18%)	Fisher’s exact test	*P* = .355
Lumbar stenosis	0	2 (9.09%)		*P* = .492
Spondylolisthesis	0	1 (4.55%)		*P* = 1.00
Revision	0	7 (31.82%)		*P* = .011

#### Oswestry Disability Index

**Figure 5.**
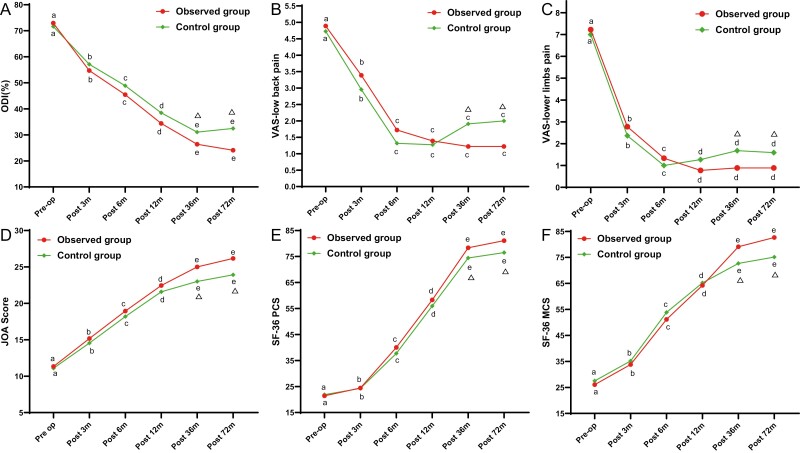
Clinical efficacy of pain and functional scores over time postoperatively between and within the 2 groups. (**A**) ODI scores over time. (**B** and **C**) The PCS and MCS of SF-36, respectively. (**D**) The changes of JOA over time. (**E** and **F**) The changes in low back pain and lower limb pain assessed by the VAS. JOA, Japanese Orthopedic Association; SF-36, the short form health survey-36; PCS, Physical Health Score; MCS, Mental Health Score; ODI, Oswestry Disability Index; VAS, Visual Analog Scale. a-e, alphabetical labeling of time point differences (comparison within the group), as long as there are the same letters at 2-time points, it means that there is no significant difference between the 2-time points ( *P* > .05), otherwise, different letters at 2-time points mean the difference is significant (*P* ≤ 0.05). Δ, significant difference in comparison between the 2 groups (comparison between groups).

The mean ODI of 72.96 ± 8.32% and 71.66 ± 8 .09% in the observed group and control group before treatment decreased to 54.70 ± 9.50% and 57.11 ± 9.19% 3 months post-op (*P* < .001), 45.44 ± 8.40% and 48.90 ± 8.36% 6 months post-op (*P* < .001), 34.39 ± 5.93% and 38.46 ± 7.19% 12 months post-op (*P* < .001), 26.39 ± 5.08% and 31.07 ± 5.34% 36 months post-op (*P* < .001), and 24.11 ± 5.58% and 32.49 ± 7.65% 72 months post-op (*P* < .001), respectively. There were no significant differences between the 2 groups at each follow-up except at the last 2 follow-ups (Fig. 5A).

#### Visual Analog Scale of Pain

The mean VAS of lower limbs declined from a baseline of 7.22 ± 0.73 in the observed group and 7.00 ± 0.81 in the control group to 2.78 ± 1.00 and 2.36 ± 1.00 at 3 months post-op (*P* < .001), and even further to 1.33 ± 0.49 and 1.00 ± 0.76 at 6 months post-op (*P* < .001), 0.78 ± 0.73  and 1.27 ± 0.88 at 12 months post-op (*P* < .001), 0.89 ± 0.68 and 1.68 ± 1.36 at 36 months post-op (*P* < .001), and 0.89 ± 0.76 and 1.59 ± 0.67 at 72 months post-op (*P* < .001), respectively. The differences between the 2 groups were statistically significant at 36 months postoperatively (*P* = .03) and at the last postoperative follow-up (*P* = .003) (Fig. 5B).

The mean VAS score for LBP in the 2 groups showed nearly the same trend as the mean VAS score of the lower limbs (Fig. 5C).

#### Japanese Orthopedic Association Score

The mean pretreatment JOA score improved significantly from baseline of 11.33 ± 2.35 and 11.09 ± 1.72 in the observed group and control group to 15.16 ± 1.76 and 14.55 ± 1.63 at 3 months post-op (*P* < .001), 18.94 ± 1.47 and 18.18 ± 2.22 at 6 months post-op (*P* < .001), 22.44 ± 1.82  and 21.59 ± 1.47 at 12 months post-op (*P* < .001), 25.00 ± 1.46 and 23.00 ± 1.63 at 36 months post-op (*P* < .001), and 26.17 ± 1.79 and 23.91 ± 2.20 at 72 months post-op (*P* < .001), respectively. Significant differences between the 2 groups were observed at 36 months postoperatively  (*P* < .001) and 72 months postoperatively (*P* = .001) (Fig. 5D).

#### The Short Form-36

The SF-36 is composed of 36 items classified into eight categories: physical function, body pain, body role limitations, emotional role limitations, mental health, social function, vitality and fatigue, and general health. Out of the items, a physical and a mental health summary score can be calculated (SF-36 physical component score, SF-36 PCS, and SF-36 mental component score, SF-36 MCS).^41^

The SF-36 PCS improved from the baseline of 21.44 ± 6.46 in the observed group and 21.88 ± 4.83 in the control group to 24.44 ± 6.74 and 24.29 ± 6.09 3 months post-op (*P* < .001), 40.02 ± 3.32 and 37.74 ± 5.10 6 months post-op  (*P* < .001), 58.28 ± 5.23 and 55.98 ± 4.64 12 months post-op (*P* < .001), 78.34 ± 5.09 and 74.42 ± 4.79 36 months post-op (*P* < .001), and 81.09 ± 5.91and 76.51 ± 4.55 72 months post-op (*P* < .001), respectively. Significant differences between the 2 groups were observed at 36 months postoperatively (*P* = .017) and 72 months postoperatively  (*P* = .009; Fig. 5E).

The SF-36 MCS also improved significantly from baseline in both groups, and the difference between the 2 groups was also at 36 months postoperatively (*P* = .001) and 72 months postoperatively (*P* = .006; Fig. 5F).

### Radiological Assessments

#### Disc Height Index

**Figure 6.**
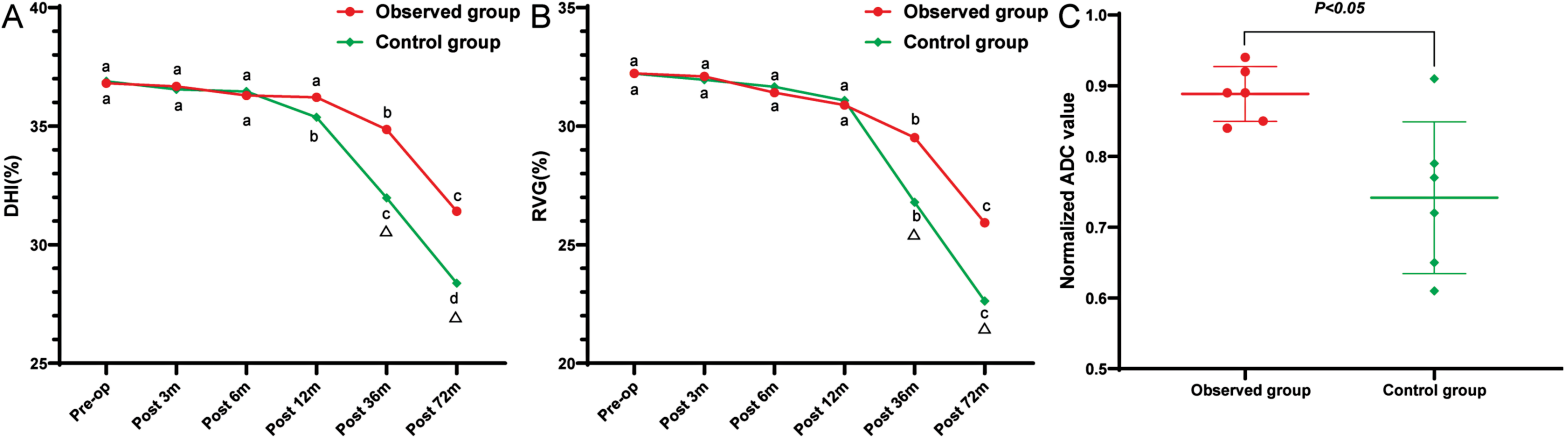
Results of radiological measurement. (A) Changes in DHI during the follow-up. (B) Changes in RVG during the follow-up. (C) The difference in ADC value at last follow-up between the 2 groups. DHI, disc height index. RVG, ratio value of the grayscale. ADC, the apparent diffusion coefficient. a-e, Alphabetical labeling of time point differences (comparison within the group). Δ, significant difference in comparison between the 2 groups (comparison between groups).

In both groups, the mean DHI of index segments tended toward a reduction, as shown in Fig. 6A. The DHIs in the observed group and control group were 36.68 ± 4.94% and 36.55 ± 4.56% at 3 months post-op, 36.30 ± 3.98% and 36.46 ± 4.35% at 6 months post-op, and 36.22 ± 4.28%  and 35.38 ± 4.45% at 12 months post-op, respectively. They were not significantly different from the baseline (36.81 ± 5.19% and 36.89 ± 4.29%). However, the DHI decreased significantly to 34.85 ± 3.94% (*P* = .099) and 31.97 ± 4.66%  (*P* < .001) at 36 months post-op and to 31.41 ± 4.37%, (*P* < .001) and 28.38 ± 4.51% (*P* < .001) at 72 months post-op compared with the baseline, and the decline in the observed group was significantly less than that in the control group.

#### Ratio Value of the Grayscale

Mean RVG in each group showed the same trend as DHI: at baseline, the mean values of RVG in the observed group and control group were 32.22 ± 6.69% and 32.22 ± 4.17%, and they were 32.10 ± 6.54% and 31.95 ± 4.44% at 3 months post-op, 31.41 ± 5.94% and 31.66 ± 4.00% at 6 months post-op, and 30.89 ± 5.66% and 31.08 ± 3.89% at 12 months post-op. The inter- and intra-group differences at these time points were minimal. However, at 36 months post-op (29.51 ± 4.38% and 26.80 ± 3.80%) and at 72 months post-op (25.93 ± 3.61% and 22.62 ± 3.72%), the RVG values decreased significantly within and between groups, as shown in Fig. 6B.

#### Pfirrmann Grades

According to the Pfirrmann classification system of T2-weighted sagittal images, there were 3 (16.67%) II, 10 (55.56%) III, 4 (22.22%) IV, and 1 (5.56%) V in the observed group and 8 (36.36%) III, 10 (45.45%) IV, and 4 (18.18%) V in the control group at the last follow-up. Pfirrmann grades in both groups differed significantly from the baseline via the Wilcoxon signed-rank test. The difference between the 2 groups was also significant at the last follow-up via the Mann-Whitney *U* test (*P* = .019).

#### Apparent Diffusion Coefficient Value

At the last follow-up, the mean ADC values of the index levels were (1.90 ± 0.09) × 10^−3^ mm^2^/s and (1.63 ± 0.20) × 10^−3^ mm^2^/s in the observed and control groups, respectively. After normalization against the ADC value of adjacent normal levels, the normalized ADC value was 0.89 ± 0.04 in the observed group compared with 0.74 ± 0.11 in the control group (*P* = .01), as shown in Fig. 6C.

#### Ultrashort Time to Echo MRI

**Figure 7.**
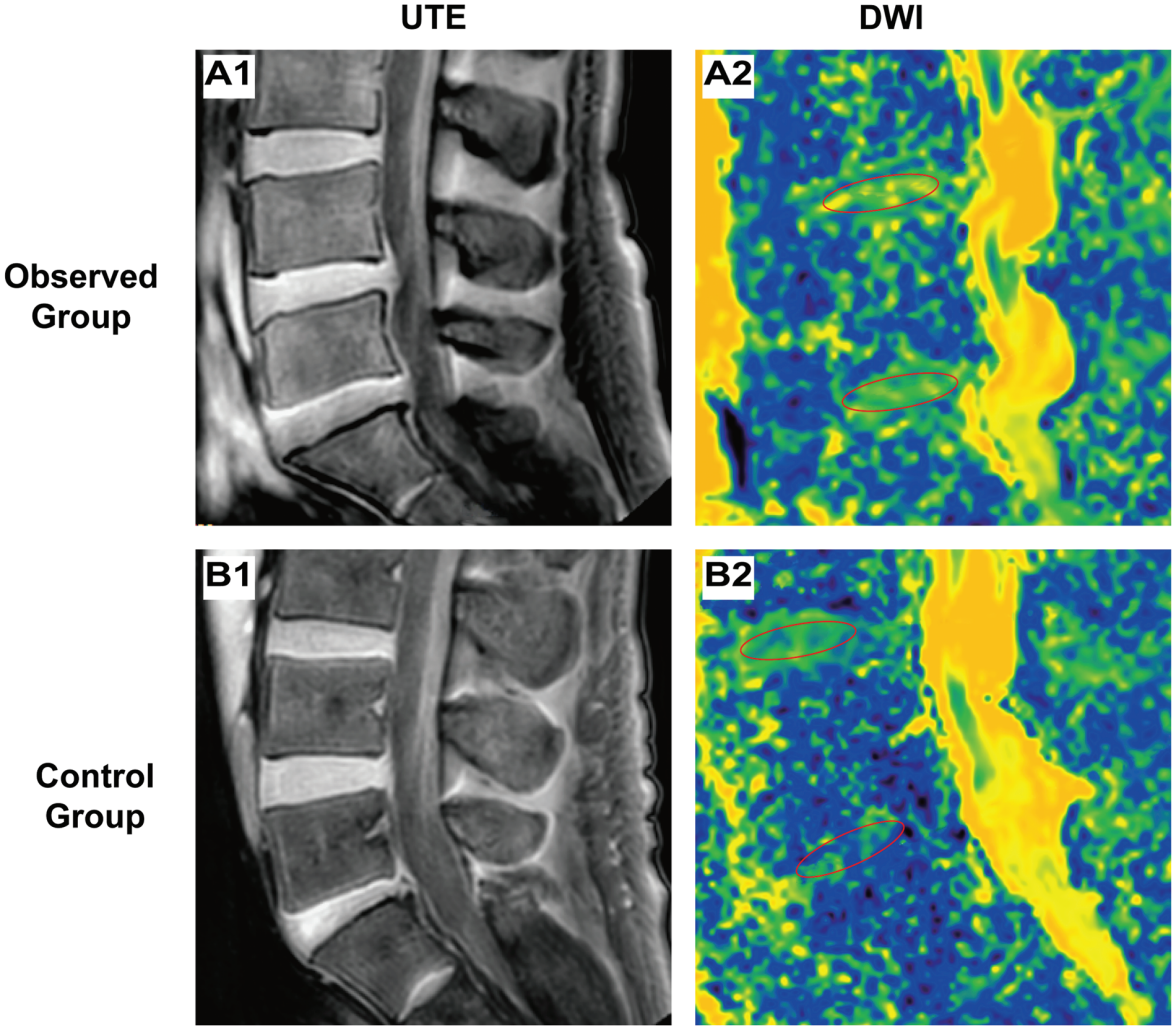
UTE MRI and DWI sequences at the last follow-up. A1 shows the relative discontinuity of the caudal CEP at the L5/S1 level. B1 shows the apparent defect of the caudal CEP at the L5/S1 level. A2 shows the corresponding DWI sequence of the same patient in the observed group and the ADC value measurement of the index level (L5/S1). B2 shows the corresponding DWI sequence of the same patient in the control group and the ADC value measurement of the index level (L5/S1). UTE, ultrashort time to echo DWI, diffusion-weighted images ADC, apparent diffusion coefficient.

Based on UTE MRI assessment, a total of 24 CEPs (cephalic and caudal) from 12 IVDs (6 in each group) were evaluated; 4 out of 12 (33.33%) and 7 out of 12 (58.33%) CEPs in the observed and control groups had defects, but the difference was not significant (*P* = .219) (Fig. 7).

## Discussion

In the present study, we first conducted a prospective cohort trial to examine the safety of discectomy followed by autologous discogenic cell transplantation in the treatment of LDH and the long-term efficacy of discogenic cells for the treatment of IVDD to demonstrate the prophylactic effect of discogenic cell transplantation to avoid secondary disease for up to 6 years after discectomy.

The most important issue that we focused on is the safety of cell transplantation. In our study, 7 (31.82%) participants in the control group underwent revision due to accelerated degeneration of the index disc, which included 4 (18.18%) disc reherniations, 2 (9.09%) lumbar stenoses, and 1 (4.55%) spondylolisthesis. The ratio of revision after discectomy in the long term was in agreement with previous reports.^42,43^ Outside of these complications, only a few AEs reported by patients were determined to be related to the procedure. The majority of these were due to post-treatment pain that resolved with conservative care. No neoplasms or heterotopic ossifications were observed on imaging at the site of cell transplantation, and no patient developed new neoplastic events after the procedure. The incidence of lumbar instability in the observed group was lower than that in the control group.

Compared to the patients treated by discectomy alone, patients who underwent autologous discogenic cell transplantation followed by discectomy gained the same clinical improvements in terms of pain and functional scores within 1 year post-treatment, but these scores improved significantly better at the last 2 visits up to 3 and 6 years after receiving the treatment. These results demonstrate that discectomy, as expected, substantially reduced the patients’ disability and pain. The trend of the sum score continued to improve in the patients whose treatment was supplemented by cell transplantation, while the control group did not sustain continual improvement in the long term.

In contrast to patient-reported outcomes, the radiological results (such as DHI and RVG) decreased gradually during the whole follow-up. Compared with baseline in both groups, the DHI and RVG of the index discs did not change significantly during the first year of follow-up, but both parameters decreased significantly at the last 2 visits (*P* < .05). Meanwhile, comparison between groups at the last 2 follow-ups showed that both DHI and RVG in the observed group decreased less than those in the control group (*P* < .05). Autologous discogenic cell transplantation slowed the decreased rate of IVD height and water content.

In addition, although 6 patients in each group underwent DWI to calculate the ADC value of index levels and adjacent normal levels at the last follow-up, the mean normalized value was higher in the observed group (88.83 ± 3.87%) than in the control group (74.17 ± 10.74%), which is in accordance with the trend of RVG. These patients also underwent UTE MRI to assess the morphology of the CEP. Although the number of CEP defects in the observed group was smaller than that in the control group, the difference was not significant. All these radiological results initially demonstrate that discogenic cell transplantation may have a potential role in retarding further degeneration after discectomy in the long term.

Discectomy is temporarily effective in eliminating symptoms arising from nerve root compression, but it does not replenish the disc lost from herniation or decelerate the speed of progression of disc degeneration. Consequently, revision (such as lumbar fusion) would inevitably be conducted in advance because of secondary diseases, including reherniation, spondylolisthesis, spinal stenosis, and ASD.^8^

Cell therapy could potentially compensate for the shortcomings of discectomy by restoring the normal function of degenerated discs. This biological treatment has been demonstrated by a large number of animal studies in vivo or in vitro as a promising technique in recent decades and has gradually progressed toward clinical translation.^44,45^ Cell therapy is a chronic process, and it cannot relieve clinical symptoms for disc herniated patients with severe neural compression.

Therefore, our hypothesis was that if we combine the surgical technique and cell transplantation, we would achieve complete neurological decompression to relieve the symptoms and decelerate disc degeneration to avoid secondary disease after discectomy.

In our study, we collected the patients’ own disc tissue, which is usually abandoned as waste during discectomy, and we cultured the cells to reach a sufficient number for transplantation without concerning autologous IVD tissue unavailability or cell inaccessibility, which are considered hurdles by many scholars.^46,47^ Meanwhile, this sampling method could avoid another surgical procedure to harvest tissues for transplanted cells, such as bone marrow and adipose tissue, which could lead to donor-site morbidity and patient fear of accepting this technique.^48^ Nevertheless, not all IVDD is combined with LDH and requires discectomy, so we had the challenge of finding alternatives to autologous discogenic cells for these patients. Additionally, the autologous approach has the benefit of reducing the risk of immunogenicity and avoiding ethical problems, although the environment within the IVD is immune-deprived.

In our study, both groups gained significant improvement immediately after the operation, without a significant difference between the 2 groups within the first year. These results were undoubtedly due to the complete neural decompression of the decectomy, but the maintenance of the efficacy and the difference in revision at 36 months or even 72 months of follow-up could be attributed to discogenic cell transplantation. The better results of radiological assessment in the observed group could further demonstrate that discogenic cells may have the potential for degenerative disc repair.

One fundamental mechanism would be that progenitor cells or stem cells in the disc tissue is activated via GMP culture and maintains their regenerative ability. Since 2007, Risbud et al first reported that degenerated human discs contain skeletal progenitors.^49^ In 2010, Blcanco first isolated stem cells from degenerative discs.^34^ Our previous study^29^ also isolated NP-derived stem cells from degenerated NP and revealed that NP-derived stem cells possess the same characteristics as bone marrow derived mesenchymal stem cells (BM-MSCs) in regeneration ability. Our other study^30^ also demonstrated the existence of NP-derived mesenchymal stem cell (NP-MSCs) in both degenerative and nondegenerative NP tissue, thus providing evidence for the presence of stem cells in degenerated NP and revealing the potential regeneration ability of discogenic cells. Other previous studies also described the presence of stem cells and progenitor cells in the tissue of the IVD.^31-34,49,50^

Another possible mechanism may be related to the interaction among the 3 kinds of discogenic cells after transplantation. In our study, AF and CEP tissues were not deliberately removed in the process of tissue separation to retain the 3 kinds of cells. In 2000, Okuma et al^51^ conducted coculture of NP cells and AF cells using the same medium and demonstrated that coculture of the 2 cell types stimulated proliferation of each. In 2016, Wang et al^31^ also used the same method of acquisition and culture of NP cells, AF cells, and CEP cells to study the differences in the biological characteristics of these and bone marrow-derived mesenchymal stem cells. Many other related studies also applied the same method of harvesting and culturing these 3 kinds of cells or NP cells alone to examine their biological characteristics and differences.^52-54^ In fact, most studies cannot separate NP tissue from AF tissue or CEP tissue completely in the process of tissue isolation under a dissecting microscope or even the naked eye, especially in degenerated discs that have vague boundaries between the NP and AF. In addition, no study thus far has identified NP cells, AF cells, and CEP cells through simple morphological observation. Based on this, we believe that the transplanted cells contain 3 kinds of cells. In our future study, we will further focus on the cellular types and characteristics.

The maintenance of biomechanical function and integrity of the disc is independent of the metabolic balance of NP cells, AF cells, and CEP cells and their paracrine effects to stimulate endogenous cells or transplanted cells to produce a neomatrix. Theoretically, the reinsertion of endogenous cells into the disc is more advantageous than exogenous cells to adapt to the hostile milieu of the disc.^18^ Moreover, many studies^32,55-57^ have demonstrated that not only does the NP tissue contain stem cells but also AF and EP tissue. Overall, transplanting discogenic cells seem to have an absolute advantage over other single-type cell transplantations in the treatment of IVDD.

We have also hypothesized the following mechanism. Based on the existence of stem cells in the disc, the cultured discogenic cells are reinserted into the index disc. These sufficient cells may contain a certain amount of stem cells and play their own repair role, including NP cells synthesizing extracellular matrix, AF cells repairing defects, CEP cells repairing injuries, and finally maintaining the structural and functional integrity of the intervertebral disc through the interaction of the 3 cells. On the other hand, disc degeneration is a natural biological process related to age, and even when performing cell transplantation to such a disc with prevention in mind, the progression of disc degeneration inevitably follows its natural course. However, transplanting autologous discogenic cells has the potential to retard the traumatic acceleration of disc degeneration after discectomy.

In this study, we not only used traditional measurement methods, such as intervertebral disc height and intervertebral disc grayscale but also used quantitative methods, such as the ADC value, to evaluate IVDD. Because of the small sample size and individual differences among patients, DHI, RVG, and normalized ADC values were selected to compare the degree of IVDD between the 2 groups. Pfirrmann classification, as a qualitative analysis, is still the mainstream method to evaluate IVDD in clinical practice worldwide. However, this classification system is very subjective and ambiguous due to the lack of a quantitative index, and it cannot be used to assess emerging cell-based therapies forIVDD.^58^ Magnetic resonance imaging (MRI), an accurate and noninvasive imaging technique, has made great progress in clinical diagnosis. Quantitative imaging has also received more attention mainly because of the relative objectivity in structure change detection, including T1rho imaging, T2 mapping, T2* mapping, diffusion-weighted imaging (DWI), diffusion tensor imaging, and chemical exchange saturation transfer.^59-63^ These recently developed quantitative MRI techniques are useful for detecting some imperceptible biochemical changes within the discs.^64^ However, the sensitivity and accuracy of these methods remain controversial. Kumar’s and Mochida’s studies,^20,21^ both of which were clinical trials to assess the efficacy of cell-based therapies for degenerative disc disease, used ADC mapping from DWI to determine the water content of treated discs and confirmed that the ADC value was more reliable to assess subtle changes in water content. Therefore, at the last follow-up, our study also attempted to use the ADC value to compare disc hydration between the 2 groups, and the results were consistent with traditional methods. Our future research will be conducted to explore the potential advantages of DWI in the assessment of disc degeneration.

Our study was the first to apply the UTE technique to evaluate the morphology of CEPs to indirectly assess the long-term efficacy of cell therapy in the treatment of IVDD. As described previously, the intervertebral disc is a 3-structure complex, cartilaginous endplates play an important role in the function and homeostasis of the disc by serving as a mechanical stabilizer as well as a pathway for nutrient transport, and its structural and compositional changes are closely related to disc degeneration.^65^ UTE MRI, a novel imaging approach, can assess MRI signals from short-T2 cartilaginous endplates that are not detected on conventional T2W MRI, thus complementing the limitations of conventional T2W MRI in assessing the integrity of lumbar discs and their clinical relevance.^66,67^ Many in vitro and in vivo studies have demonstrated a significant association between the presence of CEP defects and disc degeneration.^40,68,69^ Therefore, the evaluation of CEP abnormalities by UTE MRI can provide a subtle reference for IVD degeneration. UTE MRI is also regarded as an important tool to select appropriate patients for biological therapies of disc repair in addition to predicting individuals at high risk for IVDD.^70^ Regretfully, our study lacks preoperative UTE imaging data in each group, and there has not been a definite index or grading system of UTE imaging to assess the degree of disc degeneration. However, our report aims to raise awareness of UTE as a unique imaging biomarker to provide complementary information to that provided by conventional T1W or T2W MRI techniques, which we hope will contribute to the accuracy of disease diagnosis and the science of treatment.

At present, we should give more attention to defining the precise and optimal cell number required for functional regeneration, which is also an issue specified in a variety of studies.^25-28,71,72^ The harsh microenvironment within the disc is a potential limitation of cell-based regeneration and deteriorates in the process of degeneration. Transplanting a large concentration of cells could compete for already limited nutrients, potentially inducing more cell death and senescence and further skewing the imbalance toward degeneration.^73^ In contrast, insufficient cells cannot withstand the hostile milieu to keep viable and function ideally. Thus, our study chose a treatment amount of 10^6^ based on the experience of the above studies.

Another crucial issue we should pay attention to is cell leakage, which may result in adverse effects, such as osteophyte formation.^74,75^ Annular puncture plus discectomy defects have been demonstrated to induce degeneration if the transplanted cells fail to function well.^76,77^ However, in our study, intradiscal puncture was traditionally adopted to deliver cells without carriers or scaffolds, and no patients showed AEs related to cell leakage upon imaging examination. The primary cause of cell leakage may be the high IVD pressure with contact AF. The initial discectomy in the observed group created an “artificial cavity”, although it introduced a defect of AF at the same time, which greatly reduced the internal pressure when transplanting discogenic cells. In addition, we applied a 22-gauge puncture needle through the opposite side of the discectomy and stayed for a few minutes to maintain more cell retention, all of which can decrease the risk of cell leakage. Another mechanism is that the AF cells in the transplanted cells could home to the defective site to repair AF-like endogenous cells as well as mesenchymal stem cell homing for disc regeneration according to the studies of Wangler and Grad.^78,79^

However, with a relatively small sample size, which may lead to a certain deviation, caution must be applied, as the generalization of our results might be limited. Given that the lack of a control group and long-term follow-up are the main limitations in most of the clinical studies to date, we conducted this controlled, long-term clinical trial to further verify the real efficacy of intradiscal cell transplantation and achieved favorable outcomes.

## Conclusion

Discectomy combined with autologous discogenic cell transplantation to treat symptomatic LDH produced encouraging results in this controlled study. The results also showed a lower incidence of AEs and revision, substantially reduced pain, and increased function. Radiological analysis showed an initial trend of beneficial outcomes favoring cell transplantation. Surgery in conjunction with biological therapy can be regarded as a novel treatment concept to achieve symptom relief and prevention of secondary disease simultaneously.

## Data Availability

The data that support the findings of this study are available upon request from the corresponding author. The data are not publicly available because of privacy or ethical restrictions.
